# A serosurvey of bluetongue and epizootic haemorrhagic disease in a convenience sample of sheep and cattle herds in Zimbabwe

**DOI:** 10.4102/ojvr.v84i1.1505

**Published:** 2017-11-14

**Authors:** Stuart J.G. Gordon, Charlotte Bolwell, Chris W. Rogers, Godfrey Musuka, Patrick Kelly, Alan Guthrie, Philip S. Mellor, Chris Hamblin

**Affiliations:** 1 Institute of Veterinary, Animal and Biomedical Sciences, Massey University, New Zealand; 2 Mailman School of Public Health, Columbia University, Zimbabwe; 3 Ross University School of Veterinary Medicine, St. Kitts, West Indies; 4 Equine Research Centre, University of Pretoria, South Africa; 5 The Pirbright Institute, Pirbright, United Kingdom

## Abstract

A convenience sample of sheep and cattle herds around the cities of Harare, Kwekwe and Bulawayo, located in the Highveld region of Zimbabwe, was used to estimate the sero-prevalence and sero-incidence of bluetongue virus (BTV) and epizootic haemorrhagic disease virus (EHDV) antibodies. A competitive enzyme-linked immunosorbent assay was used to identify serum antibodies against BTV and EHDV across three rainy seasons. The median sero-prevalence of BTV and EHDV antibodies in cattle was 62% (interquartile range [IQR]: 30–89) and 56% (IQR: 5–77), respectively. In sheep, the median sero-prevalence of BTV and EHDV was 41% (IQR: 19–63) and 0% (IQR: 0–21), respectively. Median sero-incidences of BTV and EHDV antibodies in cattle of 43% (IQR: 22–67) and 27% (IQR: 9–57) respectively were recorded. The median sero-incidence of BTV in sheep was 14% (IQR: 6–23). Based on these preliminary findings, animal health workers in Zimbabwe should continue to monitor the exposure rates of cattle and sheep to BTV and consider the possibility of strains emerging with increased pathogenicity. There are no previous published reports of antibodies against EHDV in Zimbabwe so the possibility of epizootic haemorrhagic disease existing in domestic livestock should now be considered by Zimbabwean animal health officials. Seroconversions to BTV and EHDV occurred predominantly at the end of each rainy season (March and April), which generally corresponds to high numbers of the *Culicoides* vectors. BTV isolations were made from three individual cows in two of the sentinel herds and all three were identified as serotype 3. This is the first time BTV serotype 3 has been recorded in Zimbabwe, although its presence in neighbouring South Africa is well documented.

## Introduction

Bluetongue virus (BTV) and epizootic haemorrhagic disease virus (EHDV) are members of the Orbivirus genus within the Reoviridae family and are transmitted between ruminants by biting midges of the genus *Culicoides* (Diptera: Ceratopogonidae). Twenty six distinct BTV serotypes have been recognised and seven serotypes for EHDV have been identified (Cêtre-Sossah et al. 2014; Maclachlan et al. 2015).

While bluetongue (BT) remains endemic in many tropical and subtropical areas, where the vector population is abundant, this disease was previously considered to be exotic in Europe, with only a few sporadic outbreaks in Cyprus, Greece and the Iberian Peninsula prior to 1998 (Musuka & Kelly 2000). However, several different BTV serotypes have since been recorded in a number of Mediterranean regions and in 2006 the first BTV cases were identified in north-western Europe in domestic ruminants (Orłowska et al. 2016). Epizootic haemorrhagic disease (EHD) has been endemic in North America since 1955 affecting mainly white-tailed deer and, more rarely, cattle and sheep. EHDV has been isolated from epidemics in Australia, south-east Asia and Africa, and more recently on Reunion Island and in countries surrounding the Mediterranean Basin (Cêtre-Sossah et al. 2014; Maclachlan et al. 2015).

Higher mortalities from BT and EHD tend to occur in areas where the diseases are not endemic. Cyprus and Spain have recorded mortality rates up to 70% and 75%, respectively, in BT outbreaks in sheep (Gambles 1949; Manso-Ribeiro et al. 1957). During an EHD outbreak in Japan in 1959, 10% of the cattle infected with the Ibaraki strain of EHDV serotype 2 died (Thevasagayam 1998). BT is an important endemic disease of ruminants in Zimbabwe with the occurrence of the BTV being previously documented (Blackburn, Searle & Phelps 1985; Jorgensen, Halliwell & Honhold 1989; Mushi et al. 1990; Musuka & Kelly 2000). Mortality rates from BT have reached over 33% in sheep in Zimbabwe (Musuka 1999). No clinical cases of EHD have been reported in Zimbabwe to date and there are no published data on the sero-prevalence or sero-incidence of antibodies against EHDV in ruminants in the country (Gordon 2010; Musuka 1999). Although one study has isolated BTV from *Culicoides imicola* and *Culicoides magnus* (Gordon et al. 2015), the vector competence of other *Culicoides* species in Zimbabwe has not been documented and needs further investigation.

The aim of this study was, therefore, to contribute further information on the epidemiology of these two orbiviruses in Zimbabwe by determining the sero-prevalence and sero-incidence of BTV and EHDV in cattle and sheep in select ruminant and sentinel herds established around three Zimbabwean Highveld towns. Some of the viruses, isolated from blood samples from the animals in the sentinel herds that had seroconverted, were also identified.

## Materials and methods

### Identification of sampling herds

**FIGURE 1:**
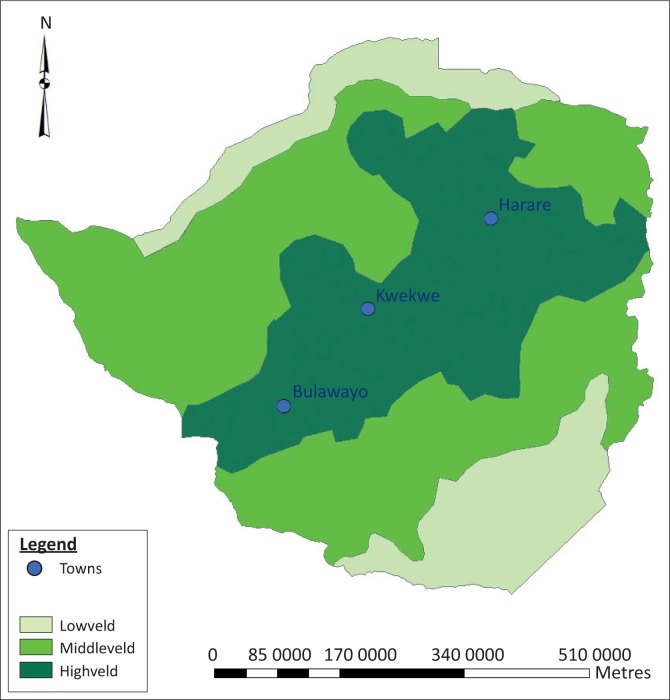
The location of the three Highveld cities in Zimbabwe around which the sampling herds were established.

Blood samples collected from cattle and sheep were used to determine the sero-prevalence and sero-incidence of BTV and EHDV on six selected farms located in the central Highveld plateau of Zimbabwe, adjacent to the cities of Harare, Kwekwe and Bulawayo (Figure 1). The climate in these regions favours *Culicoides* vectors. Farms were selected on the basis of age (> 1 year), herd size (> 8) and owners’ cooperation and were typical of the Zimbabwean Highveld, having similar soil, vegetation and agricultural practices (Gordon 2010; Gordon et al. 2015; Musuka 1999). The cattle and sheep on these premises had no history of prophylactic immunisation against BTV or EHDV, which meant that subsequently detected antibodies against these viruses would have been derived from natural challenge.

### Detection of antibodies against bluetongue virus and epizootic haemorrhagic disease virus to determine sero-prevalence

Venous blood samples (5 mL) were collected in ethylenediaminetetraacetic acid (EDTA) vacutainers from a total of 137 cattle and 72 sheep on the participating farms at the start of the three rainy seasons (1999/2000; 2000/2001; 2001/2002). Plasma, separated by centrifugation from these blood samples, was assayed by competitive enzyme-linked immunosorbent assays (c-ELISA) for BTV antibodies (Afshar et al. 1987, 1997; Anderson 1984) and EHDV antibodies (Thevasagayam et al. 1995).

### Detection of antibodies against bluetongue virus and epizootic haemorrhagic disease virus to determine sero-incidence

Animals that were negative for antibodies against BTV and/or EHDV by c-ELISA at the beginning of each rainy season were used to establish the sentinel herds. Sentinel herds consisted of a total of 44 sheep and 65 cattle. Four sentinel herds, two of cattle and two of sheep, were established on farms near Harare. Two sentinel herds, one of cattle and one of sheep, were established outside Kwekwe and two similar sentinel herds were established in the Bulawayo area.

Whole blood was collected in EDTA vacutainers every 2 weeks throughout each rainy season (November–April). Plasma was separated by centrifugation and assayed for antibodies against BTV and EHDV, using the c-ELISA methods described above. No further samples were collected from animals once they had seroconverted.

### Virus isolation and serotyping

The centrifuged red blood cells from the EDTA blood samples collected 2 weeks before sentinel animals seroconverted to either BTV or EHDV were washed and lysed following methods described by Hamblin et al. (1992) and Musuka and Kelly (2000). The chance of isolating free virus was improved by assaying the blood samples collected before animals seroconverted. Virus isolations were made by intravenous inoculation of 11-day-old embryonated hens’ eggs (IAH, UK) and by intracerebral inoculation of 1- to 2-day-old suckling mice (CD-1, UK) using techniques described by Gordon et al. (2017). The viruses isolated were typed using virus type-specific antisera and BHK-21 cells in microtitre plates using neutralisation test methods modified from Hazrati and Ozawa (1965).

### Statistical analysis

Data were described using simple descriptive statistics. The non-parametric data have been presented as medians with interquartile ranges (IQRs). The Shapiro–Wilk test was used to test the data for normality, while a Kruskal–Wallis test was used to test the effect of season on the median prevalence of BTV and EHDV. Stata IC v12 (College Station, TX, USA) was used to perform all analyses, with levels of significance set at *p* < 0.05.

## Results

### Bluetongue virus and epizootic haemorrhagic disease virus sero-prevalence and sero-incidence

The results generated by c-ELISA for the 137 cattle samples showed that the small per-farm sample size (12; IQR: 10–21) resulted in a large between-farm variation in the BTV and EHDV sero-prevalence (Table 1). The highest BTV and EHDV sero-prevalence was recorded for samples collected during the 2000/2001 season. However, there was only a significant difference recorded between the sero-prevalence of antibodies to EHDV in cattle over the three seasons (*p* = 0.043). The median sero-prevalence of BTV and EHDV in the cattle sampled across the three rainy seasons studied was 62% (IQR: 30–89) and 56% (IQR: 5–77), respectively.

**TABLE 1:** The sero-prevalence of antibodies against bluetongue virus and epizootic haemorrhagic disease virus in cattle in selected Zimbabwean Highveld herds across three rainy seasons.

Season	Number of farms	Number of cattle bled	Number seropositive for antibodies against BTV	Median sero-prevalence of BTV antibodies (%)		Number seropositive for antibodies against EHDV	Median sero-prevalence of EHDV (%)	
				Median	IQR		Median	IQR
1999/2000	3	54	23	40	0–66	19	10	0–62
2000/2001	3	48	38	100	62–100	36	92	58–100
2001/2002	3	35	28	62	20–78	11	38	0–56
All years	-	137	89	62^a^	30–89	66	56^b^	5–77

BTV, bluetongue virus; EHDV, epizootic haemorrhagic disease virus; IQR, interquartile range.

a , No significant differences between seasons (*p* = 0.638);

b , significant difference between seasons (*p* = 0.043).

The results from the 72 sheep also showed that the small per-farm sample size (12; IQR: 11–13) resulted in a large between-farm variation in the BTV and EHDV sero-prevalence (Table 2). The sero-prevalence of BTV antibodies in sheep was highest in the third season (2001/2002), but the difference between years was not significant (*p* = 0.513). The median sero-prevalence of BTV and EHDV over the three seasons was 41% (IQR: 19–63) and 0% (IQR: 0–21), respectively.

**TABLE 2:** The sero-prevalence of antibodies against bluetongue virus and epizootic haemorrhagic disease virus in sheep in selected Zimbabwean Highveld herds across three rainy seasons.

Season	Number of farms	Number of sheep bled	Number seropositive for antibodies against BTV	Median sero-prevalence of BTV antibodies (%)		Number seropositive for antibodies against EHDV	Median sero-prevalence of EHDV (%)	
				Median	IQR		Median	IQR
1999/2000	1	12	2	17	-	0	0	-
2000/2001	2	24	10	41	27–54	1	4	0–9
2001/2002	3	36	14	58	20–79	8	0	0–57
All years	-	72	26	41^a^	19–63	9	0^b^	0–21

BTV, bluetongue virus; EHDV, epizootic haemorrhagic disease virus; IQR, interquartile range.

a , No significant differences between seasons (*p* = 0.513);

b , no significant differences between seasons (*p* = 0.687).

The nine sentinel cattle herds, established over the three rainy seasons, comprised a total of 65 cattle with a median of 9 (IQR: 5–14) cattle per farm (Table 3). The small sentinel herd sample size resulted in a large between-sentinel herd variation in the sero-incidence of BTV and EHDV. Median sero-incidences for BTV and EHDV in cattle of 43% (IQR: 22–67) and 27% (IQR: 9–57), respectively, were recorded.

**TABLE 3:** The sero-incidence of bluetongue virus and epizootic haemorrhagic disease virus in cattle in selected Zimbabwean Highveld herds across three rainy seasons.

Season	Number of farms	Total number in sentinel herds	Number seroconverted against BTV	Median sero-incidence of BTV (%)		Number seroconverted against EHDV	Median sero-incidence of EHDV (%)	
				Median	IQR		Median	IQR
1999/2000	3	27	17	67	0–100	10	27	0–86
2000/2001	1	11	4	36	-	1	9	-
2001/2002	3	27	10	43	22–50	12	33	25–57
All years	-	65	31	43^a^	22–67	23	27^b^	9–57

BTV, bluetongue virus; EHDV, epizootic haemorrhagic disease virus; IQR, interquartile range.

a , No significant differences between seasons (*p* = 0.459);

b , no significant differences between seasons (*p* = 0.459).

The six sentinel sheep herds, established over the three rainy seasons, comprised a total of 44 sheep, with a median of 9 (IQR: 7–11) sheep per farm. Only one sheep seroconverted against EHDV and seven sheep converted against BTV across all three seasons. The median sero-incidence of BTV over the three seasons was 14% (IQR: 6–23).

### Viral serotyping

Virus isolations were made from three individual cows from two of the farms where the sentinel cattle were located during the 2001/2002 season. The virus type, determined by neutralisation assays for each isolate, was BTV serotype 3. No EHDV isolations were made.

## Discussion

Sheep and cattle in the selected herds in this study were commonly exposed to BTV, concurring with previous studies in Zimbabwe. Musuka and Kelly (2000) recorded sero-prevalences of antibodies against BTV in sheep and goats of up to 100% in the north-east of Zimbabwe while Jorgensen et al. (1989) reported an overall sero-prevalence of 71% in indigenous goats at 25 farms across Zimbabwe. Prevalence rates of BTV antibodies, ranging from 64% to 84%, have been reported in sheep and goat populations in South Africa (Gerdes 2004). In North Africa, BTV sero-prevalences in Algeria were found to be only 14% in sheep and 29% in cattle (Madani et al. 2011). In 455 calves sampled in Kenya, BTV antibodies were found in 94% of these animals (Toye et al. 2013). Cattle rarely show clinical signs of BT, however, and are, therefore, not vaccinated in Zimbabwe (Musuka & Kelly 2000). It should be noted, however, that the strain of BTV serotype 8, which recently emerged in Europe, appears more pathogenic in cattle than is usually the case for BTV (MacLachlan & Guthrie 2010) so the situation in Zimbabwe may change. Consequently, animal health officials in Zimbabwe should continue to monitor the exposure rates of cattle to BTV and consider the possibility of emerging BTV strains with increased pathogenicity.

Cattle sentinel herds recorded high sero-prevalences and sero-incidences of antibodies against EHDV in the selected Highveld populations in Zimbabwe. Antibodies against EHDV were detected in 64% of calves tested in Kenya (Toye et al. 2013) and in 38% of cattle and sheep sampled on Reunion Island (Desvars et al. 2015). The low sero-prevalence and sero-incidence rates against this disease recorded in sheep suggest that cattle, which are likely to be the preferred hosts of *Culicoides* vector species, as in other countries, may be a more natural host of this virus in Zimbabwe, compared to sheep. Whereas EHDV can cause a fulminant haemorrhagic disease syndrome in white-tailed deer, similar infections of ruminant livestock are usually subclinical or clinically inapparent. Nonetheless, it is now widely accepted that EHDV infections in some circumstances have resulted in disease in cattle (Maclachlan et al. 2015). Maclachlan et al. (2015) reported strains of EHDV serotypes 2, 6 and 7 as the apparent recent cause of a BT-like disease syndrome of cattle in northern and southern Africa, Reunion Island, North America and the Mediterranean Basin, including Algeria, Israel, Jordan, Morocco, Tunisia and Turkey. Affected cattle in North Africa and Israel exhibited BT-like disease signs when infected with EHDV serotype 7 (MacLachlan & Guthrie 2010). Ibaraki disease, an acute febrile disease in cattle caused by a variant of EHDV serotype 2, was first reported in the central and western parts of Japan in 1959 (Inaba 1975). The host and virus factors that may lead to the expression of EHD in cattle remain, however, undetermined (Maclachlan et al. 2015).

In Zimbabwe, the sero-prevalence of antibodies against EHDV was, however, lower than the sero-prevalence of BTV antibodies in both cattle and sheep, suggesting that domestic ruminants in this country are less susceptible to EHDV than BTV, or that the vector species of *Culicoides*, which transmit EHDV, are different to those that transmit BTV in Zimbabwe. Venter (2015) classified *C. imicola* as the most important vector of BTV to livestock in South Africa. In the Sudan, Mellor, Osborne and Jennings (1984) isolated BTV only from *C. imicola* while EHDV isolates were made from *Culicoides schultzei* group midges. Lee (1979) also reported the isolation of EHD group viruses from *C. schultzei* collected in Nigeria. Gordon et al. (2015) recorded *C. imicola* as the most abundant species trapped in Zimbabwe and only recorded very low numbers of *Schultzei* group midges.

The highest prevalence of both BTV and EHDV in cattle was detected during the second rainy season (2000/2001). This was a season which recorded above-average rainfall (Department of Meteorological Services, Belvedere, Harare, Zimbabwe). During each rainy season, most seroconversions to the BTV and EHDV occurred towards the end of the season. Mellor (1990) states that this delay reflects the increase in vector numbers and the viraemia, which develops in the infected ruminants, providing a source of infection for further vectors. Large populations of *Culicoides* vectors have been reported previously in Zimbabwe in areas with high average annual rainfall and summer temperatures (Gordon et al. 2015; Musuka et al. 2001). Large catches of *Culicoides* have also been recorded in the warm, summer rainfall areas of South Africa (Venter 2015; Venter, Nevill & Van der Linde 1997). Coetzee et al. (2012) state that BT is commonly reported in South Africa in late summer in areas with high rainfall and after good rains. Rainfall data would need to be utilised in future studies to demonstrate the association between rainfall, *Culicoides* numbers and the sero-incidence of BTV and EHDV in Zimbabwe.

In this study, the only BTV serotype identified was serotype 3. While this serotype has not been previously reported in Zimbabwe, Coetzee et al. (2012) reported that serotypes 1–6 are commonly encountered in neighbouring South Africa. Sghaier et al. (2017) recently identified serotype 3 in Tunisia while Desvars et al. (2015) attributed BTV-3 as the cause for a clinical outbreak in Merino sheep on Reunion Island. Previous studies have identified BTV serotypes 1, 2, 8, 10, 11, 12, 15, 16 and 18 from *Culicoides* vectors in Zimbabwe, although these studies were also restricted to samples from the Highveld region (Blackburn et al. 1985; Gordon et al. 2015). As yet, no data exist on the distribution of BTV or EHDV serotypes in the Middleveld or Lowveld plateaus of Zimbabwe.

## Conclusion

These results report on the sero-prevalence of BTV and EHDV in selected ruminant herds on the Highveld of Zimbabwe. Most seroconversions to BTV and EHDV occurred in March and April towards the end of each rainy season, which coincides with the peak abundance of *Culicoides* vectors. The BTV serotype isolated and identified in the blood samples from sentinel herd animals, prior to seroconversion, was serotype 3 which, while not reported previously in Zimbabwe, is commonly encountered in South Africa. Furthermore, BTV serotyping in all Zimbabwean studies to date has only been with samples collected in Highveld locations making it difficult to evaluate their national distribution. While the findings in this study are somewhat dated, no subsequent work has been conducted in Zimbabwe on these orbiviruses, highlighting the need for further research on the distribution of BTV and EHDV in Zimbabwe. There have been no previous published reports of EHD in shown so these findings have shown the presence of antibodies against EHDV in domestic livestock in another African country.
